# Efficient Care Committees

**Published:** 1915-12-11

**Authors:** 


### Efficient Care Committees.

Acting on the principle that if a thing is worth doing at all it should be done as thoroughly as possible, the Somerset County Council has been mobilising all the forces at its disposal in an endeavour to secure an adequate return for all the money which is being devoted to anti-tuberculosis measures in the county. A well-conceived step in this direction was the recent meeting at Weston-super-Mare, which was called in order to explain the progress, the objective, and the needs of the tuberculosis campaign in the county. Dr. Savage, the county medical officer, stated that about £12,000 a year was being spent, but that the County Council were prepared to spend £16,000, in order to develop the scheme in full. The provision for the treatment of and the searching for tuberculous persons in Somerset has been planned on an adequate scale, but there is one gap which voluntary effort might well be called upon to fill—namely, the formation of Care Committees. As Dr. Savage pointed out, it would be a bad day for the country when every bit of philanthropic work had to be supported by ratepayers' money. The main object of the above-mentioned meeting was the formation of a really efficient Care Committee for the Weston division, and we look for a notable success as one result, and the creation of widespread interest in the formation of Care Committees as another.

